# Concise Review: Mesenchymal Stem Cells for Functional Cartilage Tissue Engineering: Taking Cues from Chondrocyte‐Based Constructs

**DOI:** 10.1002/sctm.16-0271

**Published:** 2017-02-08

**Authors:** Andrea R. Tan, Clark T. Hung

**Affiliations:** ^1^ Columbia UniversityNew YorkNew YorkUSA

**Keywords:** Chondrogenesis, Adult stem cells, Tissue regeneration, Mesenchymal stem cells

## Abstract

Osteoarthritis, the most prevalent form of joint disease, afflicts 9% of the U.S. population over the age of 30 and costs the economy nearly $100 billion annually in healthcare and socioeconomic costs. It is characterized by joint pain and dysfunction, though the pathophysiology remains largely unknown. Due to its avascular nature and limited cellularity, articular cartilage exhibits a poor intrinsic healing response following injury. As such, significant research efforts are aimed at producing engineered cartilage as a cell‐based approach for articular cartilage repair. However, the knee joint is mechanically demanding, and during injury, also a milieu of harsh inflammatory agents. The unforgiving mechano‐chemical environment requires tissue replacements that are capable of bearing such burdens. The use of mesenchymal stem cells (MSCs) for cartilage tissue engineering has emerged as a promising cell source due to their ease of isolation, capacity to readily expand in culture, and ability to undergo lineage‐specific differentiation into chondrocytes. However, to date, very few studies utilizing MSCs have successfully recapitulated the structural and functional properties of native cartilage, exposing the difficult process of uniformly differentiating stem cells into desired cell fates and maintaining the phenotype during in vitro culture and after in vivo implantation. To address these shortcomings, here, we present a concise review on modulating stem cell behavior, tissue development and function using well‐developed techniques from chondrocyte‐based cartilage tissue engineering. Stem Cells Translational Medicine *2017;6:1295–1303*

**Significance Statement**

Using well‐developed protocols for mechanical and chemical stimulation of chondrocytes, we have consistently fabricated engineered cartilage reaching near native properties (mechanical stiffness and glycosaminoglycan content). In this concise review, we use the knowledge gained from these efforts and apply them for cartilage tissue engineering with mesenchymal stem cells to produce functional replacement tissue.

## Introduction

Articular cartilage exhibits a poor response to injury due to the intrinsic avascular nature and limited cellularity of the tissue 1. When left untreated, a progressive loss of cartilage is followed by inadequate repair and remodeling of the underlying subchondral bone and ultimately leads to osteoarthritis (OA), the most prevalent form of joint disease 2, 3. The limitations of cartilage to repair itself, coupled with inadequate clinical strategies and rising incidence rates of OA, have compelled cell‐based therapies for sustained recovery of the functional properties of native tissue.

One distinct advantage for cartilage repair is the immunoprivileged nature of the tissue due to the lack of blood vessels as well as the dense extracellular matrix of cartilage 4. As such, cartilage allografts and allogeneic chondrocytes from cadaveric donors are used clinically in biologic repair of the diathrodial joint without need for immunosuppression measures 5, 6, 7. Our team, as well as others, has implanted engineered cartilage derived from allogeneic chondrocytes in preclinical models without immune reaction 8. However, while there has been considerable success using chondrocytes in engineered cartilage constructs, their use in clinical applications may be hampered by the limited availability of cells and limited expansion capacity. Juvenile human chondrocytes show increased matrix production and do not stimulate lymphocyte proliferation when compared to adult chondrocytes, suggesting that they are not immunogenic 9, though there are significant challenges in procuring juvenile cartilage. Differentiated adult chondrocytes may be harvested from a patient's own healthy, non load‐bearing cartilage, but associated complications include donor‐site morbidity and further tissue degeneration 10, 11. Additionally, these cells may produce tissues with inferior load‐bearing ability, as inherent stiffness is known to vary across joint surfaces 12. More often, cells are retrieved from patients undergoing treatment for OA. The aged, diseased phenotype of these cells may subsequently inhibit their ability to form functional constructs 13, 14, 15.

Mesenchymal stem cells (MSCs) are an attractive alternative to chondrocytes as they are readily available, exhibit a capacity for rapid expansion, and readily differentiate into cells from a number of mesenchyme‐derived tissues, including cartilage, fat, bone, and tendon upon the application of relevant external cues 16, 17, 18. Additionally, these cells can be isolated from a wide variety of tissues including bone marrow, muscle, adipose tissue, periosteum, and synovial membrane (summarized in 19), though differentiation potentials have been found to vary depending on the source of the cell. In particular, for cartilage tissue engineering, bone marrow‐, synovium‐, and periosteum‐derived cells have been found to possess the greatest ability for chondrogenesis 20, 21. Recently, more attention has been focused on the superior capacity of synovium derived MSCs (SDSCs) to produce extracellular matrix components similar to chondrocytes (i.e., collagen II and aggrecan) after the addition of an appropriate chondrogenesis‐promoting growth factor cocktail during expansion 20, 21, 22, 23, 24, 25, 26. In further support of the use of SDSCs, Sampat et al. showed that growth factor‐priming of cells during 2D expansion coupled with a transient application of transforming growth factor β3 resulted in mechanical stiffness approaching native immature bovine cartilage levels, with corresponding glycosaminoglycan (GAG) content 24.

The use of MSCs clinically for cell‐based therapies has already garnered some clinical success. Microfracture of the subchondral bone is a common surgical technique aimed at taking advantage of the reparative capacity of MSCs, with the idea that factors secreted in the local environment of damaged tissue recruit stem cells to the site and facilitate a repair response 27, 28. Alternative uses of MSCs in cell therapy have shown some early promise; intra‐articular injections of allogenic MSCs in patients with partial menisectomy have induced tissue regeneration and pain improvement 29. Similarly, percutaneous injections of autologous bone marrow derived MSCs into a diseased knee joint have resulted in significant cartilage growth, decreased pain, and increased joint mobility 30, 31. While promising, unfortunately, the resulting fibrous tissue formation lacks the mechanical properties and structure of articular cartilage needed to sustain the rigorous loading demands. As such, the tissue engineering paradigm of incorporating cells into biomaterial scaffolds coupled with in vitro manipulations may provide a more promising approach to repairing and restoring function of damaged cartilage.

Strategies for tissue development have largely looked at recapitulating natural cartilage development, basing the selection of exogeneous cues on those found in the native local environment. The sequence of cartilage formation begins with cellular aggregation, followed by the development of a three‐layered mesenchyme, and eventual differentiation of the outer layer cells into chondrocytes 32, 33, 34. During postnatal development, cartilage is then physiologically reorganized through tissue resorption and neoformation into a highly anisotropic structure of vertical columns and horizontal strata 35. With these developmental processes in mind, tissue engineering of cartilage offers unique opportunities for therapeutics in the repair of tissues damaged by injury or disease by combining cells, biomaterials, and exogenous stimuli (recently reviewed in 36, 37, 38) to promote tissue regeneration and functional restoration of the fledgling tissue to survive the harsh loading condition and often pro‐inflammatory milieu of the native joint 39, 40, 41, 42. These approaches are based on the premise that successful culture of engineered constructs in vitro that match the material properties of native cartilage will improve the long‐term success of the replacement tissue following implantation. Moreover, this success will be greatly influenced by the ability of the engineered cartilage to integrate with the surrounding host tissue, as poor adhesion strength can lead to potential failure sites 43.

In this concise review, we examine principles of cartilage tissue engineering that we have used with chondrocyte‐based tissues to consistently generate engineered cartilage with native functional properties and translate the knowledge acquired from those efforts to MSC‐based tissues to reproduce functional cartilage replacement.

## Mechanical Stimulation

**Figure 1:**
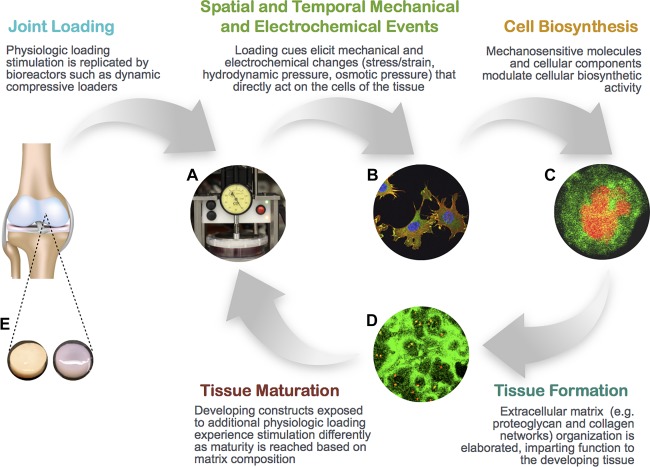
Schematic showing the role that bioreactors, which simulate aspects of the physiochemical environment of the chondrocyte, can play in modulating extracellular matrix composition and organization that gives rise to mechanical properties of the tissue. **(A)**: Deformational loading bioreactor, **(B)**: Cytoskeletal staining, **(C)**: Type VI collagen (green) staining of pericellular matrix surrounding chondrocyte, **(D)**: Extensive type II collagen network staining (green) in agarose construct, **(E)**: Native explant (left), tissue engineered cartilage (right).

A variety of physical stimuli, including osmotic loading, hydrostatic pressure, electrokinetic phenomena, stress, and strain exist in the natural joint loading environment 44. During joint and cartilage development, too, mechanical forces play an important role in joint formation with the Indian hedgehog‐parathyroid hormone‐related protein (PTHrP) feedback loop regulating the maintenance and differentiation of articular chondrocytes 45, 46. These findings have long motivated the use of mechanical stimulation such as physiologic dynamic deformational loading or sliding contact loading in chondrocyte‐based cartilage tissue engineering, resulting in improved mechanical properties and biochemical content 39, 42, 47, 48 (Fig. 1). Such loading schemes both mimic the in vivo cyclical forces at physiological levels that have been suggested to be necessary to maintain chondrocyte structure and function 49 as well as enhance the convection of nutrients through the tissue 50, 51. Additionally, the complex interplay of collagen and proteoglycan content gives rise to the unique structure‐function relationship of articular cartilage. As such, the application of physico‐chemical stimuli can modulate the structural organization 52, amount and type of extracellular matrix that gives rise to the mechanical properties of engineered cartilage tissue.

There has been evidence that similar mechanical stimulation may be beneficial for MSC‐based constructs. Results from several studies have found that physical forces can be used to modulate chondrogenesis of bone marrow derived MSCs 53, 54, 55, 56, though the timing of the application is important, suggesting a varying mechanosensitivity of the cells during chondrogenesis 57, 58, 59. For example, Huang et al. showed that loading of juvenile bovine bone marrow derived MSC constructs at early timepoints before chondrogenesis had occurred decreased functional maturation compared to non‐loaded samples even though chondrogenic gene expression increased 57. In contrast, after chondrogenesis and matrix elaboration had been initiated, loading improved the mechanical properties of the constructs. Some evidence exists that these responses, however, may be specific to stem cell source and donor. Luo et al. showed that while engineered tissues composed of either bone marrow derived MSCs or infrapatellar fat pad derived MSCs was capable of developing mechanical properties on the same order of magnitude as native juvenile tissue, dynamic compression was only beneficial to bone marrow derived MSCs 60. Further work is needed to characterize the mechanosensitivity of other stem cell sources to assess and optimize their use for functional cartilage tissue engineering.

**Figure 2:**
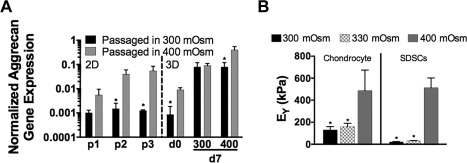
Effect of hypertonic (400 mOsm) loading on subsequent tissue development. **(A)**: 2D Expansion of human chondrocytes in higher osmolarity medium increased aggrecan gene expression normalized to glyceraldehyde 3‐phosphate dehydrogenase expression that was paralleled during subsequent 3D tissue development. **(B)**: Young's modulus (E_Y_) was significantly increased when 3D juvenile bovine chondrocyte and SDSC constructs were cultured in 400 mOsm media. *p < .05 versus 400 mOsm condition, *n* = 5/group. Abbreviations: SDSC, synovium‐derived stem cells; E_Y,_ Young's modulus.

Other relevant modes of mechanical stimulation such as hydrostatic pressure and osmotic loading have been successful at upregulating the expression of chondrogenic genes in MSC tissues 61, 62, 63, 64, 65. In particular, hypertonic loading has been found to provide a more physiologically relevant culture condition, mimicking the in vivo osmolarity of human articular cartilage (ranging from 350 to 450 mOsM depending on the zone 66, 67). In contrast, typical chondrogenic culture medium is hypotonic (330 mOsM). Indeed, physiologically relevant hyperosmotic loading during 2D expansion results in subsequently higher aggrecan expression when chondrocytes are later encapsulated in a 3D scaffold (Fig. 2A). Similarly, when hypertonic conditions are applied to encapsulated bovine chondrocytes in 3D culture, increases in mechanical properties and biochemical content are observed 63 (Fig. 2B). This beneficial effect was also observed for juvenile bovine SDSCs (Fig. 2B), producing tissues with mechanical functionality similar to native cartilage. Taken together, these studies support the use of osmotic loading regimes, either as a priming strategy or during 3D culture as a method for improvement of engineered cartilage tissue properties. Such growth factor priming strategies may also be optimized using high throughput screening measures including proteomics 68, 69.

Recently, more advanced loading regimes incorporating combinations of tensile, shear, and compressive stresses have been investigated to more appropriately mimic the in vivo loading environment 70, 71, 72. For example, Schätti et al. observed that the synergistic effect of adding a shear component to compressive loading resulted in enhanced chondrogenic gene expression of MSCs 72. Further work is needed to elucidate optimal loading regimens and parameters to control and maintain differentiation.

## Chemical Stimulation

In addition to mechanical loading to promote tissue growth, many groups have focused on the application of a range of chemical cues such as growth factors (TGF‐β3, TGF‐β1, insulin‐like growth factor (IGF), fibroblast growth factor 73, 74, 75, 76), corticosteroids 77, 78, and interleukins (IL) 40, 79, 80 for chondrocyte‐based tissue engineering as the exchange of chemical factors has been found to promote extracellular matrix (ECM) development. These same factors are potential regulators for MSCs; in fact, members of the TGFβ family as well as bone morphogenic proteins have been discovered to be the most potent inducers of chondrogenesis. Interestingly, the effects of the mechanical loading described above are amplified in, and in some cases, rely on, the presence of these growth factors 81. For example, while dynamic loading alone led to increases in GAG accumulation compared to unloaded controls, constructs exposed to TGF‐β3 alone led to much greater increase in GAG content, as well as an increase in collagen content 55. The effect of these growth factors on the transcription factor Sox9 may be the key to controlling chondrogenesis, as it has been linked to the commitment of a cell down the chondrogenic lineage. In support of the key role Sox9 plays during chondrogensis, when MSCs were manipulated to overexpress the Sox9 gene, chondrogenesis was enhanced, marked by increased proteoglycan and type II collagen deposition as well as prevention of terminal differentiation 82.

Recent studies have also examined the paracrine factors released by chondrocytes and their role in coculture systems with MSCs. Chondrocytes have been reported to secrete a range of soluble factors that promote chondrogenesis of MSCs during in vitro culture, regulating matrix remodeling, cell proliferation, and synthesis of extracellular matrix components by stem cells. For example, when combined in close proximity, mixed cell culture systems produced engineered cartilage with increased mechanical properties (Young's modulus and dynamic modulus), GAG levels, and collagen content 83, 84, 85. Interestingly, these chondrocytes were also able to decrease the deposition of collagen X 83, 86, a marker of MSC hypertrophy, perhaps by secreting PTHrP 87. Paracrine factors, therefore, may act as alternatives to growth factors that are typically supplemented in medium formulations.

It is important to note that a more thorough characterization of the influence of these two cell systems on one another is necessary to fully realize the potential of coculture systems. While a number of studies have found positive effects from chondrocytes on MSC chondrogenic differentiation 85, 88, 89, 90, other studies have found that the beneficial effects are due to the trophic role of MSCs in stimulating chondrocyte proliferation and matrix deposition 91, 92. Notably, using a xenogenic system and species‐specific gene expression analysis to determine the contribution of various cell populations to cartilage formation, Wu et al. showed that following coculture, micromass pellets contained predominantly DNA from the species of origin of the primary chondrocytes, indicating an overgrowth of chondrocytes or loss of MSCs during the culture period. Indeed, regardless of the tissue source, MSCs stimulated chondrocyte proliferation and GAG production, enhancing their potential for functional tissue repair.

## Scaffold Choice

The choice of scaffold versus acellular constructs (e.g. self‐aggregating pellets) will greatly influence the cell–cell and cell‐ECM interactions that may modulate the response of MSCs to external cues (mechanotransduction and soluble factor delivery) and vary the differentiation potential. Ideal scaffolds for cartilage tissue engineering allow for spatial control over the distribution or placement of cells to enhance chondrogenesis and also offer a template for cells to anchor onto and elaborate extracellular matrix that mimics the in vivo biomechanical environment. Toward this end, careful optimization of the materials composition, 3D structure and porosity, and biocompatibility can influence chondrogenic tissue formation. Specifically, for cartilage tissue engineering, hydrogels are particularly relevant as they are capable of retaining a high water content, mimicking the chondrogenic environment and producing homogeneous cell distributions. Some common hydrogel materials used for cartilage tissue engineering include collagen type I or type II, fibrin, hyaluronic acid, chondroitin sulfate, polyethylene glycol (PEG), alginate, and agarose 93, 94, 95, 96, 97, 98, 99. Our lab has focused extensively on agarose, a clinically‐relevant scaffold that has been used widely for maintaining the phenotype of chondrocytes (i.e., maintain a rounded morphology due to a lack of epitopes for adhesion) for basic science studies of chondrocyte biology 100, 101, 102 as well as a copolymer to support autologous chondrocyte implantation for cartilage repair in Europe 103, 104, 105. This agarose environment has proved to be beneficial for both terminally differentiated cells (e.g. chondrocytes) and MSCs (e.g. synovium‐derived stem cells), encouraging robust extracellular matrix deposition and functional response to mechanochemical stimuli similar to native tissue 24, 39, 63, 106.

However, these base polymer scaffolds can also be modified in additional ways to increase their use and potential while influencing the response of cells embedded within. Scaffolds can act as a vehicle for locally delivering growth factors or genes and also help to increase access to chemical factors for cells at the center of developing constructs. Traditionally, these cells experience nutrient diffusion limitations as engineered tissues become denser with growth in culture 107. As such, scaffolds may be modified with surface‐immobilized growth factors to increase availability to neighboring cells. In studies by Chou et al., immobilization of the growth factor TGF‐β1 produced constructs with significantly higher GAG and type II collagen production compared to exogenous delivery of the growth factor 108. Similarly, Capito et al. showed that scaffolds that incorporate nonviral IGF‐1 DNA resulted in prolonged overexpression of the growth factor throughout culture 109. Even further, the kinetics of growth factor release can be modulated depending on the method of incorporation into the scaffold such as soaking or freeze‐drying.

Hydrogels can also be modified to alter a cell's affinity for its substrate or differentiation response. For example, PEG hydrogels modified with arginine‐glycine‐aspartyl (RGD) ligands, a cell‐adhesion moiety that promotes cell attachment and adhesion, exhibited increased viability 110, 111 and enhanced cartilage‐specific gene expression and matrix synthesis in the presence of mechanical stimulation 112. Other groups have investigated coupling selected cartilaginous ECM molecules to the base polymer structure to regulate cellular differentiation. In particular, despite its absence in healthy hyaline cartilage, scaffolds containing type I collagen has been favored for mesenchymal stem cell differentiation as it has been shown to maintain chondrogenic phenotype and promote cartilage repair 96, 113. Conversely, in the presence of chondroitin sulfate, type II collagen production has been promoted and hypertrophic mineralization reduced 98, 114. Toward this end, groups have modulated the presence of these matrix molecules in the scaffold design to control chondrogenesis. For example, Nguyen et al. showed that by manipulating different combinations of PEG‐based hydrogels with ECM molecules, unique niches could be created to direct differentiation of a single MSC population toward distinct phenotypically diverse chondrocytes (i.e., superficial, middle zone, and deep zone); that is, varying ratios of polymer to ECM molecules produced different quantities of proteoglycan and collagen type II deposition representative of the different zones 115.

For clinical translation, stimuli‐responsive injectable hydrogels have garnered particular interest recently due to their minimally‐invasive delivery and ability to fill small and irregular‐shaped defect sites. These initially fluid gels can be modified and gelled in place through the addition of heat or light that react with particular cross‐linkers. Groups have used such chemistry to their advantage, modulating scaffold architecture, mechanical properties, and cellular behavior 116, 117, 118, 119, 120. However, a significant limitation exists in that they may not be as compatible with traditional functional tissue engineering strategies that require significant de novo tissue development prior to clinical application (so as to survive joint loading forces).

## Limitations With the Use of MSCs for Cartilage Tissue Engineering

While the use of MSCs for tissue engineering has shown potential, success has been variable and limited at recreating engineered tissues similar to native cartilage in terms of function or structure. These inconsistencies may point to the inherent heterogeneity associated with MSC populations in terms of cell proliferation capacity and differentiation potential 16, 121, 122, 123. For example, even when the cells uniformly express MSC markers such as CD29, CD44, CD73, CD90, CD105, and CD166, Mareddy et al. showed that there was a wide variation in cell doubling times, with fast‐growing and slow‐growing clones subsequently exhibiting altered differentiation 123. In contrast to clonal populations with rapid proliferative capacity that were mostly tripotential (capable of forming bone, fat, and cartilage phenotypes), slow‐growing clones were unipotential or bipotential. The large variation within a single population of cells and limited understanding of how to select the most potent cells for clinical application significantly challenge their use for tissue engineering.

Another challenge to cartilage tissue engineering with MSCs is how to regulate the differentiation progression, as cells may be pushed to hypertrophy, matrix mineralization, and ossification, similar to that which is in observed in the cartilage growth plate. This may be especially pertinent for clinical transplantation, as in vitro culture was found to prematurely upregulate hypertrophy‐related genes, including type X collagen, alkaline phosphatase, and matrix metalloproteinase 13 124. Subsequent implantation of these MSC pellets in ectopic sites in severe combined immunodeficient mice resulted in alterations associated with endochondral ossification rather than maintenance of a stable chondrogenic phenotype 124. Alternatively, even when chondroinductive factors induce the differentiation of MSCs into chondrocytes in vitro, the remarkable plasticity of stem cells may cause dedifferentiation following implantation or even transdifferentiation in the presence of other inductive extracellular cues 125.

Finally, the ability of MSCs to rapidly proliferate raises concerns as to whether MSCs can become tumorigenic after prolonged culture. Particularly, MSCs have been shown to be capable of secreting soluble factors to create a local immunosuppressive environment that favors tumor growth by promoting angiogenesis and preventing tumor recognition by the immune system 126, 127, 128. Though the use of MSCs in cell‐based tissue engineering and regenerative medicine is promising, further work will be needed to more fully understand the long‐term side effects of MSC‐laden constructs before clinical adoption.

## Conclusion

In summary, the use of MSCs in biomimetic environments has begun to unlock the biological potential of these repair cells. However, successful treatment for cartilage repair remains challenging, requiring a greater understanding of developmental MSC chondrogenesis and new techniques that can recapitulate the structure and function of the native tissue. Adult MSCs are a promising candidate cell source as they are readily available from a number of different tissue sources and exhibit an ability to proliferate and differentiate into desired cell types in the presence of lineage‐specific cues. Recent efforts in tissue engineering MSC‐based constructs have explored using a combination of chondrogenic stimuli coupled with innovative scaffold biomaterials to influence differentiation and proliferation, but limited studies have yielded mechanical properties matching that of native cartilage 60, 129.

Genetically modified chondrocytes or MSCs have also shown promise in the treatment of arthritis by stimulation of anabolic pathways or inhibition of catabolic pathways (reviewed in 130). Following modification by nonviral and viral vectors, MSCs can be transplanted into articular cartilage defects in vivo for sustained transgene expression level that enhances the structural features of the repair tissue. In one example of a promising use for genetically modified cells, the transplantation of cells transduced to overexpress IL‐1 receptor antagonist onto osteoarthritic cartilage explants was successful at protecting cartilage from IL‐1 induced extracellular matrix degradation 131, 132.

While these systems have yielded encouraging data, further work is necessary to understand and characterize the duration of transgene expression and the potential benefit of genetically modified cells. It also remains to be explored the subsequent in vivo response after implantation: whether the native joint will promote further chondrogenesis or if supplementation of additional growth factors is necessary to prevent hypertrophy, dedifferentiation, or transdifferentiation. Toward this end, it remains to be elucidated what the role of the in vivo environment should be: maintenance of a terminal chondrogenic phenotype or promotion of further chondrogenesis at the risk of possible proliferation. However, as temporal gene expression may not accurately reflect the production of matrix molecules that impart mechanical functionality (reviewed in 133), it is likely that for in vivo success, the most important marker by which to evaluate engineered cartilage will be the development and maintenance of mechanical properties.

**Figure 3:**
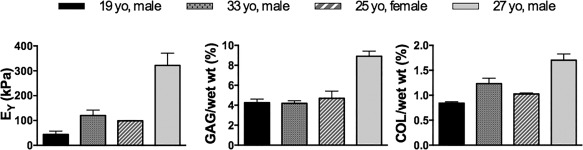
Representative data showing mechanical stiffness (E_Y_) and biochemical content (GAG; COL) of human chondrocyte constructs vary by donor. Abbreviations: E_Y,_ Young's modulus; GAG, glycosaminoglycan; COL, collagen.

Another important consideration before clinical adoption will be understanding the inherent variation of MSCs and any confounding effects of sex, age, or disease state. For example, we have seen even with human chondrocytes that inherent donor‐to‐donor differences result in variations in mechanical properties and biochemical production when these cells are used in cartilage tissue engineering (Fig. 3). We posit that the individual variations in responses and behaviors of these cells to the differentiation cues necessary to create functional cartilage may hinder efforts at defining an optimal, set protocol for producing robust tissue. One potential area of improvement in this regard may be the development of a more comprehensive library of cell surface markers found on MSCs to improve the selection of desirable cells that are more capable of producing functional tissue and help to screen potential donors. While identification of MSCs has generally relied on the presence of a core set of surface markers, there is not yet a unique set of cell surface markers or differentiation molecules delineating a specific type of MSC or its lineage potential. Work is ongoing in these efforts; for example, recently, the combination of high CD105 (Endoglin) and CD166 (cell adhesion) expression has been attributed to the chondroprogenitor phenotype 134.

Taken together, while there are still significant hurdles, with further research and development the use of MSCs to yield a clinically relevant replacement cartilage remains promising for improving the quality of life for patients with joint disease.

## Author Contributions

A.T.: developed plan for review, wrote manuscript, read and approved final manuscript; C.H.: developed plan for review, wrote manuscript, read and approved final manuscript.

## Disclosure of Potential Conflicts of Interest

The authors indicate no potential conflicts of interest.
